# As They Are Viewed by Their Fellow-Nurses

**Published:** 1919-08-02

**Authors:** 


### THE TWILIGHT OF THE GODS.

### As they are Viewed by their Fellow-Nurses.

There is a story told of the Iron Duke, when he was Prime Minister of England, as to a certain unfortunate visitor who had a bee in his bonnet. The Duke's secretary one day reported that the man was awaiting an interview. " What shall I tell him ? " he inquired. " Tell him to be damned," was the answer, and, as the secretary was disappearing, the Duke added —" but tell him politely ! "

The story is called to mind by a little paragraph in the *British Journal of Nursing* to the effect that an application to twenty-five hospital matrons asking that a representative of the R.B.N.A. might place its views on the two Registration Bills before the nursing staff, was met with a curt refusal, except by two. One of these two, who appear to have responded " politely," was represented by a single probationer, while the other contributed " an audience of seventeen, including outsiders. One wonders if this curious confession of impotence crept into the pages of the *British Journal of Nursing* by misadventure. In the most carefully regulated family circles are heard, from time to time, things that were better left unsaid. " How is the fool ? " asked an innocent boy of the young man caller. Meeting with a blank expression, the lad ventured to explain: "I heard father say that you are next door to a fool ! " It struck me when I read the unanimous rebuff of twenty-five hospitals to the blandishments of the Central Committee's Registration propagandists, that perchance the office boy in a fit of mistaken zeal had managed to blurt out the truth, and will be duly spanked when the indiscretion is discovered. One never knows. There is, of course, an alternative. An acquaintance of mine, who unwittingly played the part of entertainer to his friends, once remarked that there is no man so blind as the man who cannot see. In this he was the innocent and guileless author of a great truth. Doubtless the R.B.N.A. people think that their are none so deaf as they who refuse to hear. The combined refusal to listen, this conspiracy of invincible ignorance, may not be understood in the Intelligence Department of the Central Committee. The Duke of Wellington's bee-in-the-bonnet visitor doubtless went away wondering how on earth such a man could be the hero of Waterloo. It never by any chance occurred to him that he himself was an ass.

As I say, one never knows. I confess that I am up against a conundrum. Miss Isabel Macdonald and Miss Jentie Paterson were, apparently, the ladies who had charge of this mission, and this general refusal to listen to their oft-told tale is described as " professional apathy." Professional nausea might more accurately describe the revulsion of feeling which their reception or non-reception called forth. Do they or do they not see the point ? I have searched carefully for some clue to the mystery. I am not yet satisfied that the office boy is the culprit, for, on turning over the page, I find what is called "Word for the Week." Perhaps this also is a mistake, and that it should read " word for the weak." The " word," anyhow, is that " it is better far to go out with honour than to survive with shame ! "

I have a feeling that we are witnessing the twilight of the gods. The old order giveth place to the new. " We have no use for college kultur," exclaims Mrs. Fenwick, and therein lies the secret of the whole matter. Culture and faction are like oil and water. Mrs. Fenwick boasts of a scheme thirty years old. It is not everything, however, that improves with age.

May I commend to the consideration of these good people one of the most fascinating stories in literature. It bears on the present situation. It is by an author named Washington Irving, and entitled Rip Van Winkle.

